# The Pulsing Brain: Part II

**DOI:** 10.1098/rsfs.2025.0013

**Published:** 2025-04-04

**Authors:** Andrea Lecchini-Visintini, Emma M. L. Chung, Samantha Holdsworth, Jatinder S. Minhas, Stephen J. Payne

**Affiliations:** ^1^ School of Electronics and Computer Science, University of Southampton, Southampton, UK; ^2^ School of Life Course and Population Sciences, King's College London, London, UK; ^3^ Mātai Medical Research Institute, Tairāwhiti-Gisborne, New Zealand; ^4^ Faculty of Medical and Health Sciences & Centre for Brain Research, University of Auckland, Auckland, New Zealand; ^5^ Department of Cardiovascular Sciences, Cerebral Haemodynamics in Ageing and Stroke Medicine (CHiASM) Research Group, University of Leicester, Leicester, UK; ^6^ University Hospitals of Leicester NHS Trust, Leicester, UK; ^7^ Institute of Applied Mechanics, National Taiwan University, Taipei, Taiwan

**Keywords:** brain tissue pulsation, MRI medical diagnostics, ultrasound medical diagnostics, mathematical modelling of biological tissues, mathematical modelling of biological fluids

This issue continues the exploration of research on ‘the pulsing brain’, with contributions stemming from a Royal Society Theo Murphy scientific meeting held in June 2024 in Brighton, UK.

This part II issue reflects a peak development phase of magnetic resonance imaging (MRI) techniques, which have seen tremendous advancements in recent years. There is also an emerging focus on the circulation of neurofluids—blood, cerebrospinal fluid (CSF) and interstitial fluids (ISF)—and on the role of pulsation in driving their movement. This growing interest is motivated by recent fundamental scientific insights, frequently referenced throughout the issue, into the role of neurofluid circulation in brain physiology.

The contributions start with Lecchini-Visintini *et al*. [1] who, bringing together an interdisciplinary group of authors, offer a comprehensive review of the key areas covered in the special issues: MRI, ultrasound medical diagnostics and mathematical modelling of biological tissues and fluids.

Progress in amplified MRI (aMRI) is reviewed by Kumar *et al*. [2], who present a focused analysis of the methodology and applications of aMRI in capturing brain tissue motion. Neher *et al*. [3] utilize aMRI acquisition to generate new observations on hippocampal perfusion mechanics, providing insights into pathologies affecting this structure. Wright *et al*. [4] present an aMRI-based study illustrating changes in intracranial tissue and neurofluids dynamics under resting and exercise conditions.

In the study of fluid motion using four-dimensional (4D) Flow MRI, Dempsey *et al*. [5] investigate intracranial cardiac pulse wave velocity, providing new insights into the assessment of brain physiology. El Ahmar *et al*. [6] explore the integration of 4D Flow MRI acquisition with physics-informed image analysis to evaluate intracranial stenotic vessel segments.

Jansen *et al*. [7] present new developments in phase-sensitive diffusion tensor MRI for acquiring and reconstructing ultra-slow neurofluids’ flow in brain parenchyma.

Using DENSE MRI, McGarry *et al*. [8] illustrate the integration of DENSE MRI acquisition with patient-specific poroelastic computational models to study the driving forces behind ISF flow. Van der Plas *et al*. [9] explore the feasibility of using DENSE MRI to map brain motion in a paediatric cohort. Van der Voort *et al*. [10] conduct a simulation study investigating the robustness of DENSE MRI for assessing CSF dynamics.

In more mathematical modelling oriented contributions, Vandenbulcke *et al*. [11] present a preliminary study on integrating aMRI acquisition with computational fluid dynamics simulations of CSF flow. Koch and Mardal [12] propose a modelling framework that enables computational simulations of neurofluid flow in the brain parenchyma, providing insights into the roles of pulsation and filtration.

Together, parts I and II of the theme issue provide a comprehensive overview of techniques and recent developments aimed at advancing our understanding of intracranial pulsing dynamics. These issues are expected to foster future advancements, encouraging interdisciplinary approaches and further progress in physiology, disease progression, diagnostics and patient care.
